# Genetic alterations in primary osteosarcoma from 54 children and adolescents by targeted allelotyping

**DOI:** 10.1038/sj.bjc.6600968

**Published:** 2003-06-10

**Authors:** N Entz-Werle, A Schneider, C Kalifa, A-C Voegeli, M-D Tabone, P Marec-Berard, L Marcellin, H Pacquement, P Terrier, P Boutard, N Meyer, M-P Gaub, P Lutz, A Babin, P Oudet

**Affiliations:** 1 Service de Biochimie et Biologie Moléculaire, CHRU Hautepierre, Avenue Molière, 67098 Strasbourg Cedex, France; 2 Service de Pédiatrie Onco-Hématologie, Institut Gustave Roussy, 39 rue Camille Desmoulins, 94809 Villejuif Cedex, France; 3 Service de Pédiatrie Onco-Hématologie, Hôpital Trousseau, 26 avenue du Dr Netter, 75571 Paris Cedex 12, France; 4 Service d'Oncologie Pédiatrique, Centre anticancéreux Léon Berard, 28 rue Laennec, Lyon, France; 5 Service d'Anatomopathologie, CHRU Hautepierre, Avenue Molière, 67098 Strasbourg Cedex, France; 6 Service d'Oncologie Pédiatrique, Institut Curie, - 26 rue d'Ulm, 75231 Paris Cedex, France; 7 Service d'Anatomopathologie, Institut Gustave Roussy, 39 rue Camille Desmoulins, 94809 Villejuif Cedex, France; 8 Service d'Onco-Hématologie Pédiatrique, CHRU Caen, Avenue de la Côte de Nacre, 14033 Caen Cedex, France; 9 Laboratoire de Biostatistique et informatique Médicale, Département d'Information Médicale, CHRU Strasbourg 1 place de l'Hôpital, 67091 Strasbourg, France; 10 Service de Pédiatrie Onco-Hématologie, CHRU Hautepierre, Avenue Molière, 67098 Strasbourg Cedex, France

**Keywords:** high-grade osteosarcoma, microsatellites, paediatric population, Rb, p53, 5q21 region, 7q31 region

## Abstract

At present, the only recognised prognostic factor for primary osteosarcoma is the histological response to preoperative chemotherapy. Our study was designed to identify new diagnostic markers that could eventually have a prognostic value. A total of 54 patients under 20 years of age with primary osteosarcomas were studied while under treatment by the French Society of Paediatric Oncology OS 94 protocol. Paired normal and biopsy samples were collected. In addition, surgical resection specimens, following preoperative chemotherapy, were obtained in 13 cases. After genomic DNA extraction, an allelotyping analysis targeting microsatellites linked to Rb and p53 genes, and 9p21, 7q31 and 5q21 regions was performed. In all, 94% of the samples at diagnosis showed allelic imbalance and the biopsies were highly rearranged except for the microsatellite targeting 7q31. The same panel was highly informative at surgical resection. Microsatellites investigating Rb, p53 and the 9p21 region were particularly altered without a significant correlation with prognosis. On the other hand, the alteration of the 7q31 locus at diagnosis was significantly correlated with a worse prognosis and a new frequently altered locus, 5q21, was described. In conclusion, this panel allowed us to characterise paediatric osteosarcomas. Correlation of prognosis with the altered 7q31 region could be a useful tool and further studies are required to confirm the importance of 5q21.

Primary high-grade osteosarcoma is the most frequent malignant bone cancer in childhood accounting for about 5% of all paediatric solid tumours. It is diagnosed mainly in teenagers and mostly localised in the long bones of limbs. Anatomopathological analysis of tumour biopsy confirms only the diagnosis of high-grade osteosarcoma without any prognostic consequence (Dahlin and Unni, 1986; Huvos, 1991). In one study, the site of the primary tumour had no correlation with survival (Nagler *et al*, 2000), whereas others have reported a significant trend towards survival in the axial or appendicular flat bone high-grade osteosarcomas (Duffaud *et al*, 2000; Gadwal *et al*, 2001).

In this study, after diagnosis, the patients were treated with the OS 94 protocol, which included preoperative chemotherapy, followed by surgery and postoperative chemotherapy. In this protocol of the French Society of Paediatric Oncology (SFOP), a randomised preoperative chemotherapy was administered. The first line combined either anthracycline and high-dose methotrexate or etoposide, ifosfamide and high-dose methotrexate. After surgery, patients were classified as good (GR) or poor responders (PR) to preoperative chemotherapy according to the histological grading system established by Huvos *et al* (1977). Postoperatively, GR were treated with the same drugs and PR received a second-line chemotherapy. Assessment of histological response to preoperative chemotherapy is currently the only major prognostic tool. Thus, a clear need exists for early identification of patients that would relapse or fail to respond. Until now, the validation of prognostic diagnostic factors has been unsuccessful.

In the majority of primary osteosarcomas, the aetiology is unknown, but, for a minority, it occurs in the setting of a previous cancer, such as a hereditary mutation of retinoblastoma or an autosomic recessive mutation of p53 in the Li-Fraumeni syndrome (Huvos *et al*, 1977; Malkin *et al*, 1990; Alonso *et al*, 2001). These two genes, localised in 13q14 and 17p13, respectively, could therefore be involved in the multistep oncogenesis model of high-grade osteosarcomas. Indeed, the currently accepted model of oncogenesis corresponds to a stepwise accumulation of genomic defects.

Until now, the histological analysis at diagnosis and on surgical resection has been insufficient in explaining the oncogenesis of osteosarcomas and the worse evolution of some GR. Immunohistochemistry has allowed analysis of the expression of several protein targets, like proteins involved in cell cycle or differentiation, but with a low sensitivity (Wadayama *et al*, 1994; Ferracini *et al*, 1995; Nielsen *et al*, 1998; Maitra *et al*, 2001; Park *et al*, 2001). Likewise, cytogenetic studies of osteosarcomas have shown various complex cytogenetic changes involving some chromosomes but without any specific pattern (Mertens *et al*, 1993; Ozisil *et al*, 1994). At the molecular level, some of the biological factors proposed thus far as potential prognostic factors in osteosarcoma are p15, p16 (Nielsen *et al*, 1998; Maitra *et al*, 2001), p53 (Park *et al*, 2001), Rb (Wadayama *et al*, 1994) and c-met (Ferracini *et al*, 1995; Oda *et al*, 2000), but these studies have mainly been performed on small populations.

Allelotyping analyses by identification of microsatellite modifications could reveal directly at the DNA level the presence of chromosomal alterations and could characterise either chromosomal (allelic imbalance or AI) or microsatellite instabilities (MSIs). The presence of MSIs identifies the repair error phenotype (RER) (Karran, 1996). Although the allelotyping is a powerful and sensitive method, it does not establish whether allelic imbalances correspond to allelic gains or losses. Microsatellite studies can however allow the characterisation of some loci potentially involved in the multistep carcinogenesis, like p16, p53 and Rb genes. These were the cell cycle genes shown to be most frequently altered in osteosarcomas in previous studies, but, with controversial prognostic implications (Tsuchiya *et al*, 2000; Maitra *et al*, 2001; Park *et al*, 2001). A French working group has produced a first retrospective molecular study, showing a significant trend in the association of alteration at Rb locus and a bad prognosis (Feugeas *et al*, 1996). Up to now, the status of many differentiation and proliferation genes such as c-met or APC, which are known to be involved in other cancers have not yet been analysed in paediatric osteosarcomas. Our study, including 54 patients, was therefore designed to identify prognostic factors assessable at diagnosis in osteosarcoma by analysing the microsatellite status of 5q21, 7q31, 9p21, 13q14 and 17p13 loci. Furthermore, the results were correlated with the histological response to preoperative chemotherapy and survival.

## PATIENTS AND METHODS

### Patients

In total, 54 patients (Table 1

**Table 1:** Clinical and molecular data of the 54 patients included in our study that are in CCR1 or CCR2, in relapse, in partial remission (PR) or dead

**Patients**	**Age (years)**	**Localisation**	**Histologic response**	**Survival (months)**	**TP53**	**D7S486**	**RB1**	**D9S171**	**D5S346**	**D5S492**	**Status**
1	7	Femur	GR	76	h	h	AI	AI	h	h	CCR1
2	14	Tibia	GR	81	h	h	N	AI	N	h	CCR1
3	16	Tibia	PR	81	h	AI	h	h	h	h	CCR1
4	10	Femur	GR	77	AI	N	AI	h	N	h	CCR1
5	13	Tibia	GR	82	AI	AI	AI	h	AI	AI	CCR1
6	12	Femur	PR	83	h	N	AI	AI	AI	h	CCR1
7	15	Pelvis	PR	78	AI	AI	AI	AI	AI	h	CCR1
8	13	Femur	PR	86	AI	N	AI	h	N	h	CCR1
9	12	Femur	GR	73	AI	N	N	AI	AI	h	CCR1
10	13	Humerus	GR	85	AI	AI	AI	N	AI	h	CCR1
11	13	Tibia	GR	74	AI	AI	AI	h	h	h	CCR1
12	11	Femur	GR	86	AI	N	N	N	N	h	CCR1
13	14	Femur	PR	86	N	N	AI	N	AI	h	CCR1
14	13	Femur	GR	80	AI	h	N	AI	h	h	CCR1
15	8	Femur	GR	74	AI	AI	AI	N	N	h	CCR1
16^a^	15	Tibia	PR	65	AI	h	N	AI	N	h	Dead
17	15	Pelvis	GR	71	h	h	h	AI	AI	h	CCR1
18	Unknown	Unknown	GR	72	N	N	N	N	N	N	CCR1
19^a^	8	Humerus	Unknown	57	h	h	AI	N	AI	h	CCR1
20	17	Femur	PR	22	h	h	N	AI	AI	AI	CCR2
21	9	Femur	GR	35	h	h	h	h	AI	AI	CCR2
22	4	Tibia	GR	54	h	N	AI	h	N	h	CCR1
23	17	Femur	PR	57	AI	h	AI	N	N	h	CCR1
24	16	Humerus	PR	71	AI	h	AI	N	AI	h	Dead
25	12	Tibia	PR	65	N	h	N	N	N	h	Dead
26	9	Femur	GR	78	N	N	h	AI	AI	AI	CCR1
27	12	Femur	PR	69	AI	N	N	h	N	N	CCR1
28	13	Femur	GR	39	AI	N	AI	AI	N	h	CCR1
29	13	Femur	GR	19	AI	AI	AI	h	N	h	Dead
30	10	Femur	PR	39	h	h	AI	AI	AI	h	CCR1
31	12	Femur	GR	28	h	N	AI	h	AI	h	CCR1
32	9	Tibia	GR	75	AI	N	AI	h	N	h	CCR1
33	14	Femur	GR	32	AI	h	h	AI	AI	h	CCR1
34	17	Fibula	GR	32	AI	h	h	AI	AI	AI	CCR1
35	20	Humerus	GR	52	h	h	h	h	AI	h	CCR1
36	13	Fibula	GR	77	N	h	h	h	N	N	CCR1
37^a^	15	Femur	PR	19	AI	h	AI	N	AI	h	Dead
38	7	Femur	GR	54	AI	h	AI	AI	h	N	CCR1
39	15	Tibia	PR	69	AI	N	h	h	h	h	CCR1
40	10	Humerus	GR	77	AI	AI	h	h	AI	h	CCR1
41	15	Femur	PR	11	h	h	N	AI	h	h	Relapse
42^a^	9	Femur	PR	7	h	AI	h	AI	AI	AI	PR
43	13	Tibia	GR	28	AI	N	AI	AI	h	N	CCR1
44	9	Mandibular	PR	7	N	N	AI	AI	AI	h	Dead
45	7	Femur	GR	30	AI	h	h	h	h	N	CCR1
46	14	Femur	GR	30	h	h	AI	AI	h	h	CCR1
47	13	Femur	PR	12	AI	N	h	N	h	h	CCR1
48	12	Femur	PR	54	h	N	AI	AI	h	h	CCR1
49^a^	14	Tibia	PR	32	AI	N	h	h	AI	AI	CCR1
50	13	Femur	GR	38	h	h	N	AI	N	N	CCR1
51	16	Femur	PR	24	h	N	AI	h	AI	h	CCR1
52	16	Tibia	PR	24	h	AI	h	N	AI	h	PR
53^a^	15	Femur	GR	3	AI	N	AI	h	AI	AI	CCR1
54	10	Femur	PR	12	AI	AI	h	AI	AI	h	Dead
					29 AI=83%	11 AI=34%	27 AI=71%	23 AI=66%	26 AI=58%	h	Dead

a The results concerned homozygous (h) or heterozygous patients (with AI or normal status (N)). ^a^Metastatic patients. (GR=good responders (30); PR=poor responders (23).

) with primary high-grade osteosarcoma were included in our molecular study. All were treated following the guidelines of the OS 94 protocol between November 1994 and October 2001. Six patients had a metastatic disease at diagnosis (patients 16, 19, 37, 42, 49 and 53). The population consisted of 30 GR and 23 PR, according to the histopathological grading reported by Huvos *et al* (1977). Only one patient was not assessable. The GR patients were defined with a necrosis cutoff of 95% or more. This population comprised 30 boys and 24 girls. The median age at diagnosis was 13 years old (range from 4 to 20 years). The initial tumour localisation consisted of 31 femurs, 12 tibias, two fibulas, five humerus, one mandibular and two pelvises. In one case, these data were not assessable. At the end of our study, among these 54 patients, 42 are in first continuous complete remission (CCR1), two in second continuous complete remission (CCR2), two in partial remission, one in relapse and seven died. All deaths were because of progressive and metastatic osteosarcomas, highly resistant to chemotherapy. No toxic death was observed in this cohort. The median survival was 57 months (range from 3 to 86 months).

### Sample collection for molecular analysis

Biopsies from 54 patients were collected at diagnosis, fresh–frozen and stored at −80°C. The biopsies were histologically characterised by a pathologist. In addition, samples from the surgical resection, following the preoperative chemotherapy, were collected in 13 cases. The histological grading was made in these 13 cases according to the previously defined necrosis cutoff. For one patient, we obtained the relapse tissue and for another, the metastatic specimen. Paired normal tissue was obtained from peripheral blood samples conserved on Whatman paper.

## METHODS

### 

#### DNA extraction

Tissue and blood paired DNA were purified as described in Miller *et al* (1988) and Schneider *et al* (2000). Tumour and blood genomic DNA concentrations ranged from 50 to 400 ng *μ*l^−1^ and from 1 to 10 ng *μ*l^−1^, respectively.

#### Microsatellite analysis

Six microsatellites were analysed on paired normal and biopsy DNA. They were informative for p53 (*TP53*), localised in the Rb gene (*RB1*, intron 20), in the 9p21 (*D9S171*) and 7q31 (*D7S486*) regions and at the 5q21 locus. *D5S346* and *D5S492* at 5q21 were centromeric and telomeric, respectively, in a region containing the APC gene among others. Sequences of primers were obtained from the genomic database (http://www.ncbi.nlm.nih.gov/genemap99 and http://www.gdb.org). Genomic DNA from both the paired samples (10 ng) was amplified by PCR in a total volume of 50 *μ*l using 1.2 U of *Taq* polymerase and 4 pmol of both forward and Cy5 labelled reverse primers. PCR was carried out in an Omnigen Hybaid Thermocycler (Hybaid Ltd, Ashford, UK) using the following protocol: 7 min at 95°C, 35 cycles of 1 min at 95°C, 1 min at 55°C (*D9S171*, *D5S346*, *D5S492*, *TP53*) and 53°C (*RB1*, *D7S486*) and 1 min at 72°C, followed by 5 min at 72°C.

#### PCR product analysis

**Figure 1:**
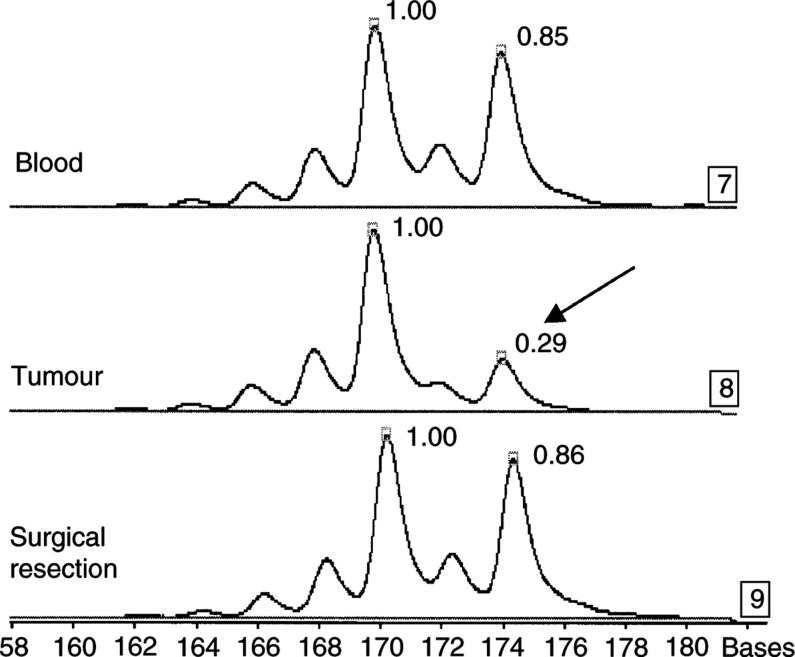
Allelic imbalance (AI). Genomic DNA was extracted from blood, tumour biopsy at diagnosis and tumour of surgical resection (patient 1). PCR of D9S171 was separated by using a sequencing analyser (ALF Pharmacia). The microsatellite was informative and shows a change in the ratio between the two amplified alleles comparing blood and paired tumour biopsies (black arrow). This patient normalised his surgical tumour sample, there is no change in the ratio between the two amplified alleles comparing blood and paired surgical tumour.

After electrophoresis on denaturing urea gel on an ALF (Automated Laser Fluorescent) sequencer (Amersham-Pharmacia Biotech, Uppsala, Sweden), amplified fragments were directly detected and quantified by the Alfwin Fragment Analyser software package. A modification of the allele ratio in paired tumour and leukocyte DNA is described as AI (Figure 1

). The presence of additional peaks is described as MSI. This technique is highly reproducible and sensitive. As determined previously in our laboratory, a cutoff of 20% was used to identify significant AI (Schneider *et al*, 2000). Each alteration was confirmed at least twice by PCR.

### Statistics

All data were computed using SPSS 9.0 for windows (SPSS, Inc. Chicago, IL, USA). The survival function was estimated using Kaplan–Meier test. The log rank to compare survival and the *χ*^2^ test were used to analyse correlation between the AI and subgroups of patients.

## RESULTS

**Figure 2:**
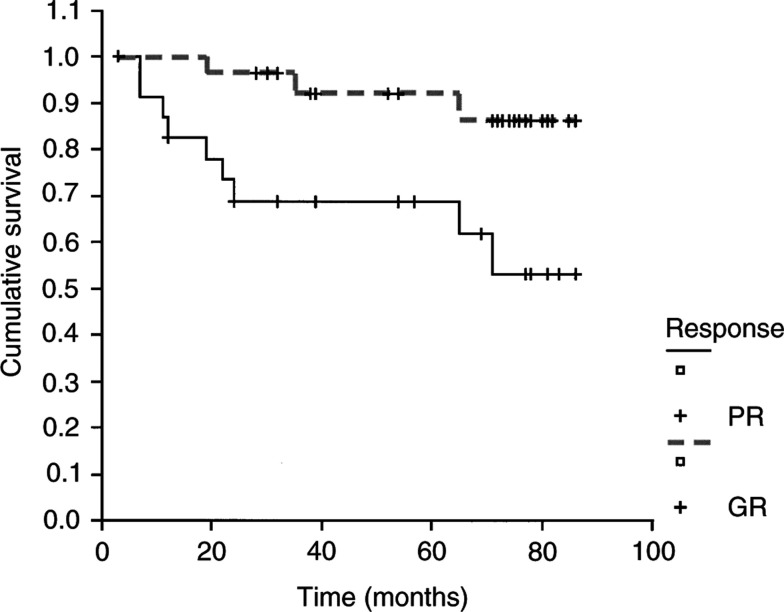
Kaplan–Meier EFS curves for the GR (- - - - -) and PR (——) with a statistical significance (*P*=0.01).

We obtained paired blood and tumour biopsies at diagnosis from 54 children. In addition for 13 out of 54 cases, we also collected surgical resection fragments, allowing a comparison between pre- and postchemotherapy. Characteristics and survival of the entire group are detailed in Table 1. Overall, the event-free survival (EFS) rate at 5 years was 80%. Histological diagnosis showed, in all cases, high-grade osteosarcomas. In all, 30 GR and 23 PR were identified. All the surgical resections were histologically reviewed and all PR patients had less than 95% tumour necrosis. The statistical analysis of these two groups revealed a significant difference of EFS at 5 years (92 and 69%, respectively, *P*=0.01) (Figure 2

), associating PR with a worse prognosis. Microsatellite analysis was realised on all samples using six couples of primers. Primer sets were informative for the p53 gene, localised in Rb or in 9p21, 5q21 and 7q31 regions. The last three loci contain, in addition to genes of unknown functions in oncogenesis, genes involved in cell cycle and differentiation, like p16, APC and c-met.

### Frequent AI are observed in biopsies as compared to blood DNA

The allelotyping did not detect additional peaks (MSI) in our population for our set of six microsatellites. Furthermore, no MSIs were detected at 10 additional microsatellites analysed (D3S700, D3S1283, D4S3019, D4S428, D7S2455, D7S2595, D17S800, D17S1818, D20S107 and D20S855). Thus, it appears that repair error mechanisms were probably not involved in these tumours. In heterozygous patients, a change in the ratio between the two amplified alleles was frequently observed when blood and paired tumour biopsies were compared, indicating the presence of AI. In all, 94% (51 out of 54) of the patients showed at least one AI, demonstrating that our selection of six microsatellites was highly informative for identifying rearranged primary osteosarcomas at diagnosis (Table 1). Samples (three out of 54), lacking microsatellite alterations (patients 18, 25 and 36), showed, in fact, AI at two other microsatellites targeting 3p (*D3S1293* and *D3S3700*), thus confirming the presence of tumour cells. In the informative tumours, we observed a variation of the intensity of the allelic ratio ranging from 35 to 90% in comparison to normal DNA. This was consistently higher than the previously defined cutoff. These percentages confirmed the high proportion of tumour cells in our biopsies. In each biopsy, the observed allele ratio was at the same intensity for every loci analysed.

**Figure 3:**
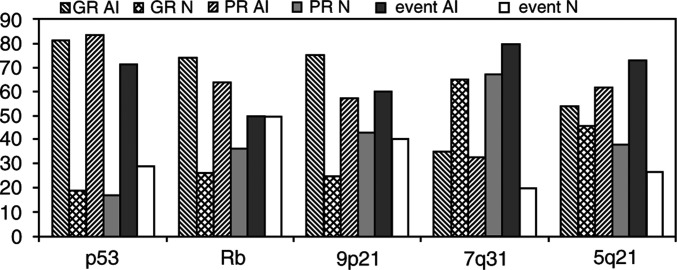
Allelic imbalance (AI) frequencies and percentages of normal (N) status at the five targeted loci in the different subgroups of patients: GR, PR and the population combining different events such as partial remission, relapse and deaths (event).

A high level of AI frequency was observed at each locus, except for the microsatellite targeting 7q31. Taking into account heterozygous loci, AI was found in 83% (29 out of 35) for *TP53*, 71% (27 out of 38) for *RB1*, 66% (23 out of 35) for *D9S171*, 34% (11 out of 32) for *D7S486* and 58% (26 out of 45) for *D5S346* and *D5S492* (Table 1). Furthermore, a slightly higher proportion of homozygous loci, than reported in the database, was observed at *D7S486* (41 *vs* 35% for *TP53*, 30% for *RB1*, 35% for *D9S171* and 17% for 5q21). At least one of the cell cycle genes, p53, Rb or locus 9p21, was altered in 88% biopsies. In order to analyse the data at the locus containing APC, we used two microsatellites flanking this region (*D5S346* and *D5S492*), and considered this locus as informative if it was heterozygous for both markers or if at least one of the two markers was modified, and the second one noninformative. In cases where both microsatellites (*D5S346* and *D5S492*) were informative, results at both sites were always identical, either both AI or both normal. In GR and PR subgroups, AI frequencies for each microsatellite did not show statistical differences (Figure 3

). In the subgroup, combining relapses, partial responses to treatment or dead patients (patients 16, 24, 25, 29, 37, 41, 42, 44, 52 and 54), a higher AI frequency was observed at *D7S486* and at *D5S3546* and *D5S492*, *but a statistical significance was found only for the 7q31 locus* (*P*=0.04) (Figure 3).

**Figure 4:**
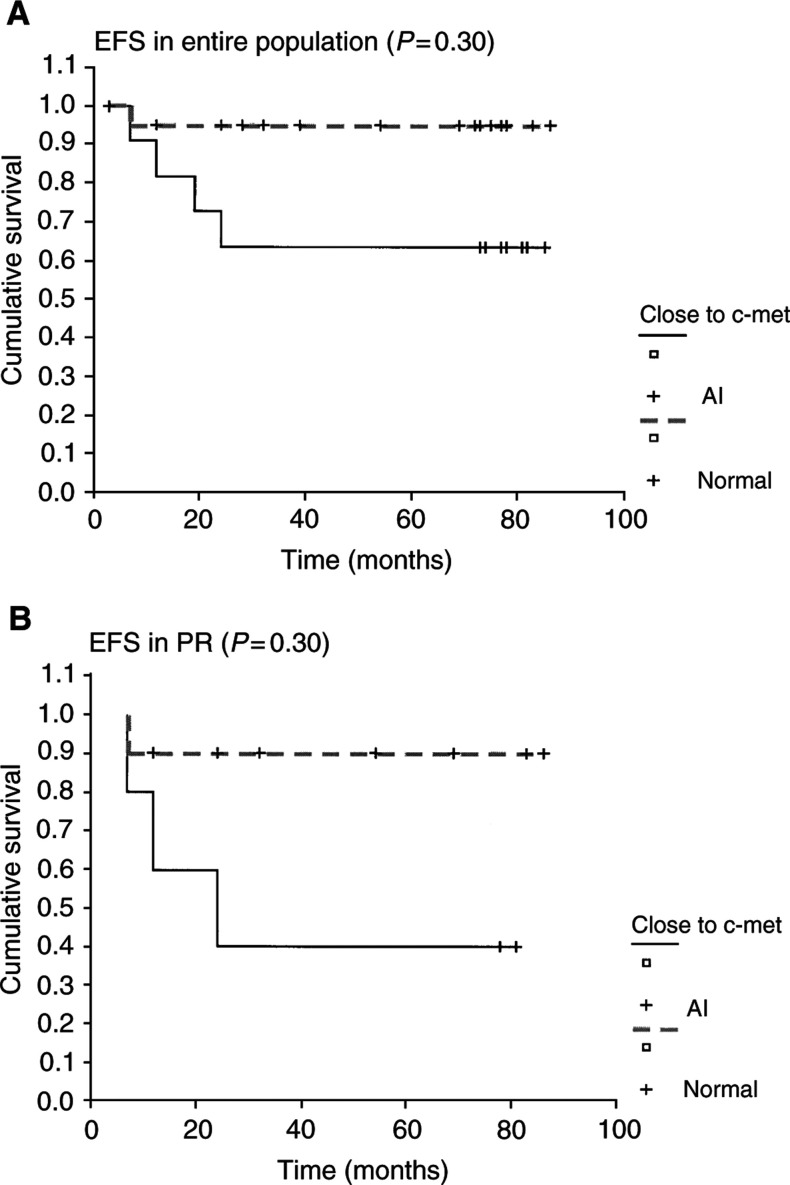
Kaplan–Meier EFS curves of the microsatellite analysis at 7q31 locus for the entire population (**A**) and for the PR (**B**) with a statistically significant association between the presence of AI and a worse prognosis (——).

**Figure 5:**
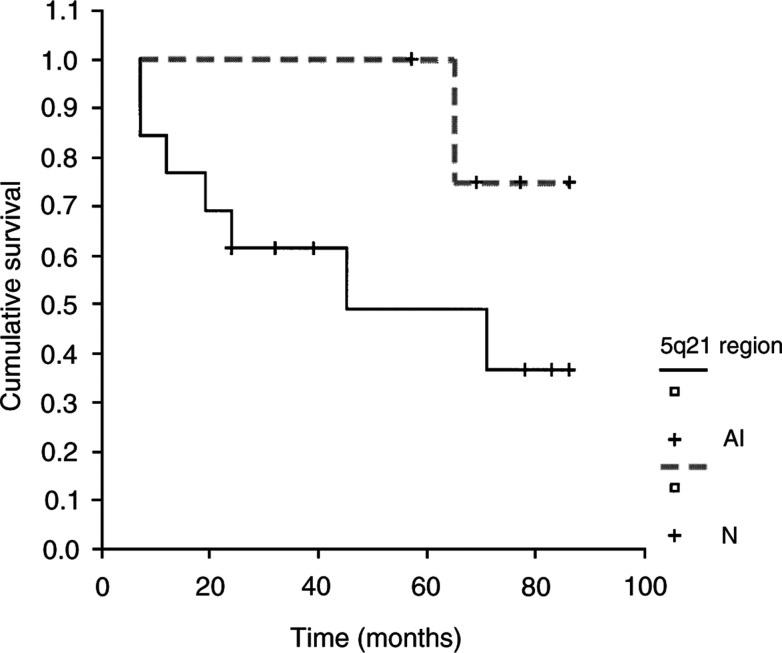
Kaplan–Meier EFS curve for the PR with AI at 5q21 region and without alterations. This statistical analysis showed a trend between the presence of AI and a worse survival.

Event-free survival was investigated for each microsatellite in the entire population. No significant differences in survival time were observed to be related to the presence of AI at *TP53*, *RB1* or *D9S171*. Interestingly, a normal status at 7q31 was associated with a significantly better EFS not only in the whole population, but also in the PR subgroup (Figure 4

) (*P*=0.03). The association of a rearrangement at markers flanking 5q21 with a bad prognosis is suggested by the curves, but the correlation is not statistically significant (*P*=0.15) (Figure 5

).

### Comparison of tumour samples at diagnosis with the surgical resection following chemotherapy

Fortunately, we were able to collect a surgical sample in addition to biopsy for 13 cases (nine PR and five GR) (Table 2

**Table 2:** Molecular analysis of the 13 patients whose normal tissue, biopsy at diagnosis and surgical resection samples were analysed

			**TP53**	**D7S486**	**RB1**	**D9S171**	**D5S346**	**D5S492**	**Status**
Patient 1	GR	Biopsy	h	h	AI	AI	h	h	CCR1
		Tumour	h	h	N	N	h	h	
Patient 3	PR	Biopsy	h	AI	h	h	h	h	CCR1
		Tumour	h	N	h	h	h	h	
Patient 4	GR	Biopsy	AI	N	AI	h	N	h	CCR1
		Tumour	AI	N	AI	h	N	h	
Patient 6	PR	Biopsy	h	N	AI	AI	AI	h	CCR1
		Tumour	h	N	N	N	AI	h	
Patient 8	PR	Biopsy	AI	N	AI	h	N	h	CCR1
		Tumour	AI	N	AI	h	N	h	
		Metastasis	AI	N	AI	h	N	h	
Patient 10	GR	Biopsy	AI	AI	AI	N	AI	h	CCR1
		Tumour	N	AI	N	N	AI	h	
Patient 11	GR	Biopsy	AI	AI	AI	h	h	h	CCR1
		Tumour	N	N	N	h	h	h	
Patient 16	PR	Biopsy	AI	h	N	AI	N	h	Dead
		Tumour	AI	h	N	AI	AI	h	
Patient 27	PR	Biopsy	AI	N	N	h	N	N	CCR1
		Tumour	AI	N	N	h	N	N	
Patient 29	GR	Biopsy	AI	AI	AI	AI	N	h	Dead
		Tumour	AI	AI	AI	AI	AI	h	
Patient 47	PR	Biopsy	AI	AI	h	N	h	h	CCR1
		Tumour	AI	AI	h	AI	h	h	
Patient 49	PR	Biopsy	AI	N	h	h	AI	AI	CCR1
		Tumour	N	N	h	h	N	AI	
Patient 54	PR	Biopsy	AI	AI	h	AI	AI	h	Dead
		Tumour	AI	AI	h	AI	AI	h	
		Relapse	AI	AI	h	AI	AI	h	

The results were homozygous status (h), AI or normal status (N). This study involved GR and PR that were in first CCR1 or dead.

). In three out of five GR, the presence of AI was still observed (patients 4, 10 and 29). All five GR patients had between 95 and 99% necrosis. However, the allotyping analysis allowed us to identify four different groups.

First, four children (patients 1, 3, 11, 49), comprising two GR and two PR, normalised their surgical resection, probably suggesting the absence of any significant tumour cell population according to our molecular detection. These four patients are alive and in CCR1. In the second group (one PR and one GR, cases 6 and 10), the alterations at 7q31 and 5q21 regions persisted, but the other AI previously identified were no longer observed. The third group concerned four patients (cases 4, 8, 27, 54), who showed the same rearrangements in both biopsy and tumour resection. For these four patients, our molecular analyses suggested an initial significant chemoresistance of the localised tumour cell population, even though one patient was classified as GR in CCR1. Finally, in the last group (patients 16, 29, 47), new microsatellite alterations appeared in comparison with biopsy, which were localised at the locus 5q21 for patient 16 (PR) and 29 (GR). Both patients died. Histological response was not correlated to the results of microsatellite analysis in any of the groups. In the relapsing and metastatic specimens, we detected the same alterations, as observed in biopsy or surgical samples, suggesting the evolution of the same tumour clone (Table 2).

## DISCUSSION

### Overall considerations about our biopsy collection

This study was designed to identify molecular prognostic factors assessable at diagnosis, as Rb and p53 genes, 9p21 and 7q31 loci and a region surrounding the APC gene. Candidate genes in 9p21 and 7q31 are p16 and c-met, respectively. Altogether, clinical and statistical data of our population appeared to be representative of the usual histological diagnoses and treatment responses in the multicentre OS 94 protocol. The main difficulty was obtaining paired blood and tumour DNA samples, mainly because of the low incidence of such sarcomas. Despite this problem, our study included 54 primary paediatric high-grade osteosarcomas, treated with the same therapeutic protocol. Previous published studies concerned either adult or paediatric populations, mixed the low- or high-grade osteosarcomas and had different therapeutic regimens (Ozisil *et al*, 1994; Patino-Garcia and Sierrasesumaga, 1997; Nielsen *et al*, 1998; Tsuchiya *et al*, 2000). To our knowledge, no other molecular study has investigated such a large homogeneous paediatric population.

Our microsatellite analysis was achieved in 54 paired blood and fresh–frozen biopsy specimens. Allelic imbalances were detected, but there were no MSI, allowing us to infer that the RER phenotype (Fearon and Vogelstein, 1990; Kinzler and Vogelstein, 1996; Hanahan and Weinberg, 2000) is not the main mechanism involved in the oncogenesis of osteosarcoma. Belchis *et al* (1996) showed MSI at the retinoblastoma locus in eight out of 18 informative osteosarcomas, but their study investigated formalin-fixed, paraffin-embedded samples, which could affect microsatellite analyses. With this panel of six microsatellites, 94% of our population showed DNA alterations at diagnosis. This high frequency recommends the choice of this sensitive and powerful method and these selected markers for routine analyses at diagnosis. Furthermore, our results showed a high intensity of allelic ratio alterations, confirming the high percentages of tumour cells in these biopsies. The clonal homogeneity of tumour samples was confirmed by the same high percentage allelic ratio intensity at each locus analysed from the same tumour.

### Analysis of results and comparison of biopsy DNA samples and paired blood DNA for the loci involved in the cell cycle

With this sensitive technique, high AI frequencies in p53, Rb and 9p21 loci were observed. These high frequencies of alterations at *TP53* and 9p21 (locus containing CDK inhibitor INKAp16) were in agreement with previous data (Nielsen *et al*, 1998; Tsuchiya *et al*, 2000; Benassi *et al*, 2001). Several studies concerning these genes have reported lower frequencies, however they used either protein level analysis or mutation detection and are therefore difficult to compare with microsatellite analysis. In contrast with these studies, we did not find any correlation between the presence of AI and prognosis (Nielsen *et al*, 1998; Maitra *et al*, 2001).

Concerning the Rb locus, the same frequency (70%) of AI was found as in the previous SFOP study (Feugeas *et al*, 1996). According to the previous report, there was no significant correlation between the abnormalities at this locus and the histological grading in our present study (Feugeas *et al*, 1996). In contrast, in our study no correlation between EFS and AI at RB1 was observed, while the previous study suggested correlation of Rb locus alteration and poor prognosis. Probably, such discrepancy could be explained by the modifications of the chemotherapy between these two protocols. In fact, we analysed a different prognostic group of patients, since in our study 30 GR and 23 PR were explored *vs* 12 GR and 19 PR in the previous one (Feugeas *et al*, 1996).

Altogether, our results, like previous data, confirmed the putative role of 9p21 region and Rb/p53 signalling pathway in osteosarcomas (Nielsen *et al*, 1998; Tsuchiya *et al*, 2000; Benassi *et al*, 2001). However, the lack of correlation between survival and the presence of alteration at those loci suggested that other mechanisms are involved in the sensitivity to chemotherapy.

### Microsatellite analysis of 5q21 and 7q31: a prognostic marker and a new probable target diagnosis

Results at the locus 7q31 and at the region containing APC suggested a striking and interesting role for these sites in osteosarcoma oncogenesis, even though the frequencies of rearrangements at the locus 7q31 were rather low (34%) (Table 1). Nevertheless, when we only considered the group comprising partial remissions, relapses and patients who died, this AI frequency reached 80%, resulting in a significant correlation with this bad prognostic subgroup (*P*=0.04). Thus, our data have identified a new region involved in the prognosis of osteosarcomas. The significant correlation between alteration of 7q31 and a worse prognosis demonstrates its prognostic role. Results obtained at this locus were in agreement with previous c-met studies, concluding that c-met could play a role in aggressive osteosarcomas (Ferracini *et al*, 1995; Oda *et al*, 2000). Even though such results should be confirmed *in a larger population* with a longer follow-up and a c-met gene quantitative analysis, the knowledge of the presence of AI at this locus could be useful in predicting GRs that will relapse and might encourage the use of kinase inhibitors in osteosarcomas treatments in future.

At the locus 5q21, the use of two microsatellites, surrounding the APC gene, increased the specificity of allelotyping at this site and allowed us to speculate that the high percentage of rearrangement could strengthen the fundamental role of this gene in osteosarcoma. To our knowledge, such results in osteosarcomas have only been described in one recent study (Monaghan *et al*, 2001), since APC has previously been known to play a role in the oncogenesis of colorectal cancer (Polakis, 1997). Furthermore, statistical analyses showed a slight trend for the presence of AI at 5q21 correlating with the poor response to chemotherapy, in agreement with a trend towards a worse prognosis in case of AI at 5q21. However, a larger cohort would be required to confirm this. In addition, further studies would be necessary to define precisely the status of this gene or the presence of mutation.

### Allelotyping surgical resection specimens as additional tool for refining the histological response to preoperative chemotherapy

Since some GR are known to relapse earlier, refining the stratification of GR could help to adjust treatment. Our allelotyping of surgical resections was very informative, allowing us to characterise four subgroups that did not appear to be correlated with histological response. In the groups where AI were still detected in the surgical specimens, we identified two situation: either same alterations or new AI were observed in surgical samples compared to biopsies. The second situation suggested the presence of new cellular clones, possibly selected by chemotherapy since the reproducibility and high intensity of AI agreed strongly with the presence of a dominant cell clone at diagnosis. It should be noted that the patients who died (patients 16 and 29) had additional AI at 5q21, strengthening the role of this loci in poor prognosis contrary to the case in colorectal cancer (Polakis, 1997). In fact, only GR with persisting AI should be carefully followed since these GR could belong to the subgroup who could relapse earlier. Altogether, allelotyping at surgical resection time could complete the histological analysis and help to provide a more precise stratification of each subgroup of responders.

In conclusion, our large population of 54 paediatric osteosarcomas was investigated with five targeted loci. These five loci proved to be good diagnostic markers in at least 94% of samples and revealed a potential implication of 5q21 region containing APC. A significant correlation between presence of rearrangements at 7q31 and a worse prognosis was observed. Finally, the use of targeted microsatellite analysis at surgical resection could be an additional tool to make more precise stratification, especially in GR.
